# IgG N-Glycan Profiles in Mothers and Infants Postpartum in the Context of Maternal Obesity and Gestational Diabetes

**DOI:** 10.3390/ijms262110641

**Published:** 2025-10-31

**Authors:** Anna Farkas, Oksana Matsyura, Lesya Besh, Andras Guttman, Sandor G. Vari

**Affiliations:** 1 Horváth Csaba Memorial Laboratory of Bioseparation Sciences, Research Center for Molecular Medicine, Faculty of Medicine, University of Debrecen, 4032 Debrecen, Hungary; anna.farkas@med.unideb.hu; 2 Department of Pediatrics No. 2, State Non-Profit Enterprise “Danylo Halytsky Lviv National Medical University”, 79010 Lviv, Ukraine; omatsyura@gmail.com (O.M.); lesya.besh@gmail.com (L.B.); 3 Translational Glycomics Group, Research Institute of Biomolecular and Chemical Engineering, University of Pannonia, 8200 Veszprem, Hungary; 4 Research and Innovation in Medicine Program, Cedars-Sinai Medical Center, Los Angeles, CA 90048, USA

**Keywords:** obesity, gestational diabetes mellitus, IgG, N-glycosylation, capillary electrophoresis

## Abstract

This study explored IgG N-glycosylation pattern differences in maternal and infant serum in the context of gestational diabetes mellitus (GDM). Serum samples from 15 mother–infant pairs were collected at 12 weeks postpartum and categorized according to maternal body mass index (BMI) and GDM status. The N-glycosylation patterns of the isolated IgG pools were analyzed by capillary electrophoresis with laser-induced fluorescence detection (CE-LIF). Descriptive comparison of the relative area percentage of IgG N-glycan structures revealed differences between the groups. Comparison of the maternal and infant sialo-form/neutral-form ratio (SF/NF) of the N-glycans suggested differences between control mothers and their children, as well as between obese mothers and their children. The maternal SF/NF ratio of IgG varied between the obese and normal-weight GDM mothers. The SF/NF ratios of IgG from the infants showed variation between infants of control mothers and infants of obese mothers, between infants of obese and infants of obese GDM mothers, and between infants of GDM with normal-weight and GDM with obese mothers. The observed differences in maternal and infant IgG N-glycosylation profiles suggest potentially selective placental transfer mechanisms.

## 1. Introduction

According to the World Health Organization (WHO), gestational diabetes mellitus (GDM) is a multifactorial metabolic disorder that first manifests during pregnancy. It is associated with persistently high blood sugar levels and disturbances in the metabolism of carbohydrates, fats, and specific proteins due to insufficient insulin action [1]. GDM has short- and long-term adverse health consequences for both the mother and the offspring [2]. Although the diagnostic criteria and screening procedures for GDM vary from country to country [3,4,5], the most common way to diagnose is the oral glucose tolerance test (OGTT) conducted between 24 and 28 weeks of pregnancy [6,7]. However, since GDM can develop at any time during pregnancy, even after a negative screening test [8,9], its risk factor predictive value is relatively low, resulting in undiagnosed and untreated GDM [10]. Consequently, there is a growing need to identify biomarkers specific to GDM.

An increase in the prevalence of GDM has been attributed to factors such as a family history of type 2 diabetes mellitus (T2DM) or GDM, especially among close relatives, advanced maternal age, and overweight. A significant proportion of women of reproductive age are overweight or obese, with this number reaching 44% worldwide in 2022 [11]. Therefore, maternal obesity and its impact on the offspring have received increasing attention in recent years [12]. Based on epidemiological data, it is evident that the children of obese women are significantly more predisposed to obesity [13], hypertension, type 2 diabetes, and metabolic syndrome [14]. This intergenerational transmission of obesity and associated comorbidities highlight the need to better understand the complex mechanisms that link maternal obesity to offspring health. Although GDM can also develop in women with normal body weight, maternal obesity remains one of the major risk factors. Therefore, examining how GDM-related biomarkers differ between obese and normal-weight women may provide insights into the mechanisms of GDM development and help to identify early indicators of risk for GDM.

Proteins are essential biomolecules involved in cellular processes, and their functional diversity is largely expanded by post-translational modifications (PTMs) [15]. Among these, glycosylation is one of the most prevalent and functionally significant PTMs, playing critical roles in numerous biological processes, including cellular recognition and signaling, immune cell recognition, antigen presentation, morphogenesis, regulation of cell growth, and proper neuronal development and function—all of which are essential for maintaining extracellular matrix structure and tissue integrity [16]. Protein glycosylation is a process by which specific enzymes attach sugar residues to proteins, thereby modifying their structure and function [17]. Glycosylation can occur through different types of linkages—most notably N-glycosylation and O-glycosylation—which are highly conserved and closely associated with disease pathogenesis and progression [18]. Therefore, the glycosylation patterns of proteins are the result of the joint effects between numerous glycosidases and glycosyl-transferases [19], that are highly responsive to cellular physiological and pathological changes, therefore, can be promising biomarkers for various diseases [20,21].

Immunoglobulin G (IgG) is the most abundant N-glycosylated protein in human serum. The composition of the oligosaccharides at the conserved glycosylation site of Asn297 within the Fc region greatly influences the effector function of the molecule [22,23,24]. Two specific glycan modifications, sialylation and fucosylation, are key regulators of IgG activity. Increased sialylation is generally associated with anti-inflammatory properties, as it reduces Fcγ receptor binding and dampens effector responses, whereas decreased sialylation has been linked to enhanced pro-inflammatory activity [25]. The anti-inflammatory effect of IgG Fc sialylation has been linked to its interaction with the lectin DC-specific ICAM-3–grabbing non-integrin receptor (DC-SIGN) on myeloid regulatory cells, which induces IL-33 production and subsequently drives the expansion of IL-4–producing basophils. This cascade promotes increased expression of the inhibitory Fcγ receptor FcγRIIB, thereby raising the activation threshold of innate effector cells in response to immune complexes [22]. In addition, Fc sialylation has been shown to attenuate immediate pro-inflammatory effector functions of IgG by impairing complement-mediated cytotoxicity [26]. In contrast, the presence or absence of core fucosylation strongly affects IgG affinity for FcγRIIIa: afucosylated IgG binds more efficiently, thereby amplifying antibody-dependent cellular cytotoxicity (ADCC) [27]. Through these mechanisms, sialylation and fucosylation fine-tune the balance between pro- and anti-inflammatory signaling, ultimately shaping the outcome of immune responses. Consequently, N-glycosylation modification of IgG Fc part can influence both disease progression and remission, even serving as an effective indicator of inflammatory processes [24]. Therefore, N-glycosylation alterations of IgG may reflect an already established disease state and play an important role in the pathophysiology of diabetes [28].

Hyposialylated IgG has been implicated in obesity-induced insulin resistance through activation of the IgG receptor FcγRIIB [29]. Specific IgG N-glycan structures, namely FA2B and A2G2S2, have also been associated with metabolic parameters linked to insulin levels and insulin resistance [30]. In addition, BMI has been shown to correlate positively, albeit modestly, with the levels of agalactosylated glycans of IgG [31]. The observed decrease in galactose content, which correlates with increased BMI, may reflect the chronic “low-grade inflammation” typically associated with obesity development. The “low-grade inflammation” experienced due to visceral obesity causes easily detectable glycosylation modification changes in IgG [32]. These glycovariant antibodies transmitting pro-inflammatory signals can also harm the offspring, triggering certain inflammatory mechanisms.

The objective of this work was to provide an exploratory characterization of maternal and infant IgG N-glycosylation patterns in relation to maternal obesity and gestational diabetes mellitus (GDM) using capillary electrophoresis analysis with laser-induced fluorescence detection (CE-LIF), considering that postpartum samples may reflect the consequences of pregnancy rather than causal factors.

## 2. Results

### 2.1. Patient Characteristics

The maternal BMI during pregnancy was significantly higher in the H-O (31.36 ± 1.43; *p* < 0.0001) and GDM-O (32.49 ± 2.15; *p* < 0.0001) groups compared to the H-NW (22.75 ± 1.21) as a control group. Analyzing the gestational age, we detected significant difference between the H-O (37.96 ± 1.41 weeks) and GDM-O (39.96 ± 1.66 weeks) groups (*p* = 0.0034). Although maternal age differences between the groups did not reach statistical significance (*p* > 0.05), the observed variation may still be clinically relevant. Given the small sample size and limited statistical power, potential effects of maternal age cannot be excluded. Moreover, due to the pooled nature of the samples, no individual-level statistical analysis could be performed, and potential confounders such as maternal age could not be adjusted for the regression models. Nevertheless, maternal age differed by approximately five years between the groups, which may still be clinically meaningful given its well-established role as a risk factor for GDM. Therefore, residual confounding by age cannot be excluded when interpreting the glycosylation results. We found that the rate of normal vaginal delivery decreased to 53.34% and 73.34% in the GDM-NW and GDM-O groups, respectively, compared to 80% in the control group. These are both notable decreases, but interestingly, there was also a slight increase in the rate of normal delivery in GDM-O compared to GDM-NW. In the H-O group, we observed an unexpected increase in the proportion of vaginal deliveries (86.7%) compared with the control group (80%). The clinical parameters of the patients included in this study are detailed in Table 1.

### 2.2. Serum IgG N-Glycosylation Analysis of Mother-Child Pairs

**Figure 1:**
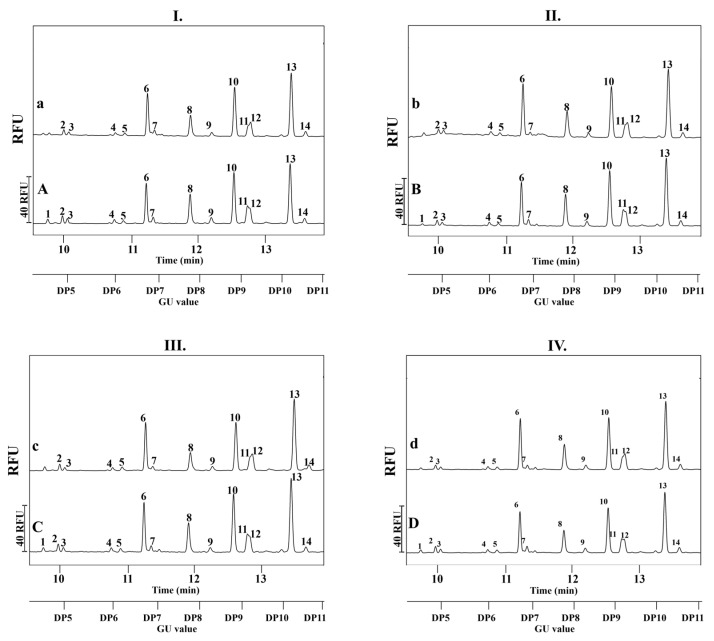
Comparison of the IgG N-glycosylation profiles of pooled serum samples collected from mother-infant pairs. (**Panel I.**) Healthy normal BMI mothers (A) and infants (a); (**Panel II.**) Obese mothers (B) and their infants (b); (**Panel III.**) Mothers with normal BMI and GDM (C) and their infants (c); (**Panel IV.**) Obese mothers with GDM (D) and their infants (d). The upper X-axis of the subfigures represents the migration time of the peaks in minutes, while the lower X-axis depicts the GU values. The relative fluorescence units (RFU) of the peaks are displayed on the Y-axis. Separation conditions: 50 cm effective length (60 cm total, 50 μm i.d.) BFS capillary with NCHO separation gel buffer; 30 kV in reversed polarity mode. Separation temperature: 25 °C; Sample injection: 1 psi for 5 s. APTS-labeled maltose injection: 1 psi for 5 s.

In the first phase of the study, IgG antibodies were partitioned from the serum samples of both mothers and infants using Protein G affinity chromatography microcolumns. The N-glycosylation pattern of these antibodies was subsequently analyzed as exploratory group-level observations to assess the impact of obesity and GDM. Please note that because the analyses were performed on pooled samples, the data are descriptive in nature, and statistical analyses at the individual level could not be performed. Representative electropherograms for each group are shown in Figure 1. N-glycan structures were included in the analysis only if their relative peak area exceeded 1% of the total glycan profile in the control group. Accordingly, a total of 14 peaks and their associated 16 N-linked sugar structures were identified across all examined maternal groups. In contrast, 13 peaks representing 15 distinct N-glycan structures were observed in the serum samples of the infant groups. The structural data and detailed information of each numbered peak are given in Table 2.

### 2.3. Serum IgG N-Glycan Profile in Relation to Maternal BMI and Gestational Diabetes

Based on the relative area percentages of the detected glycan peaks, clear differences could be observed between the maternal groups. Among the 14 peaks analyzed, only three (peaks #5, #7, and #13 corresponding to A2G2S1; FA2BG2S1 and FA2G2) showed similar values across all groups, while the others displayed group-specific variations.

**Figure 2:**
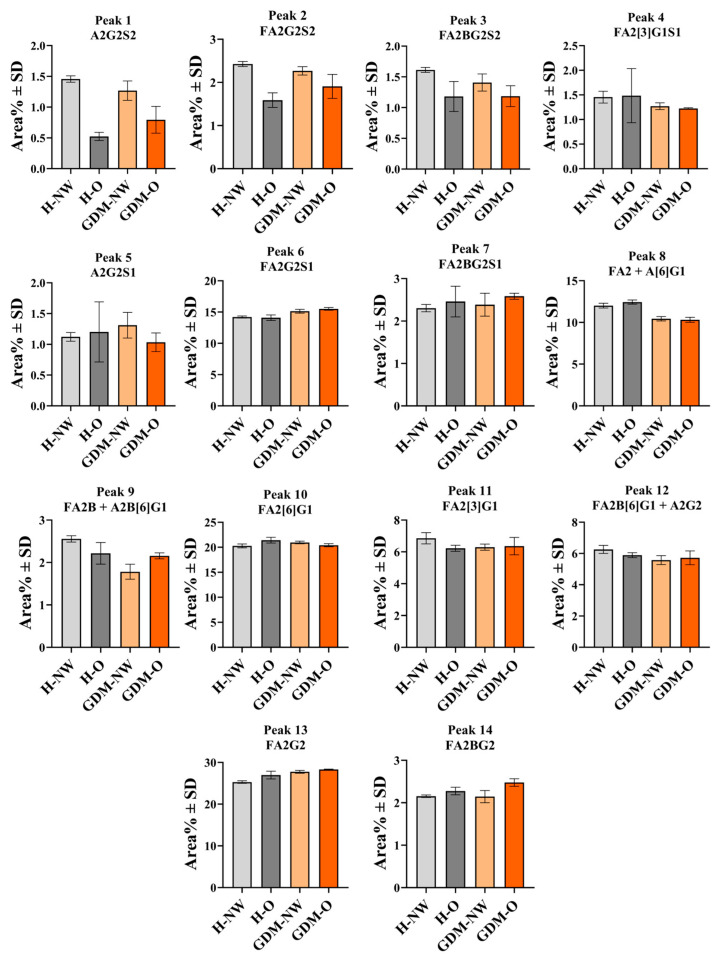
N-glycan structures detected in the H-NW (healthy normal weight), H-O (healthy obese), GDM-NW (GDM with normal weight), and GDM-O maternal groups (GDM with obesity). Each group consisted of pooled samples of 15 individual specimens. The columns represent mean relative area percentage values ± SD. The mean and SD were calculated from three independently prepared replicates of the pooled sample, with each replicate measured three times (technical replicates), to assess measurement reproducibility. Differences between groups are presented descriptively.

The H-NW and H-O groups could be distinguished by four peaks corresponding to A2G2S2, FA2G2S2, FA2BG2S2, FA2[6]G1. On the other hand, the H-NW and GDM-NW groups could be distinguished from each other based on two peaks representing four Asn-linked glycan structures (peak #9—FA2B + A2B[6]G1, and peak #12—FA2B[6]G1 + A2G2). The H-NW and GDM-O groups showed the most pronounced differences, with seven peaks exhibiting distinct relative abundances (peak #1—A2G2S2, peak #2—FA2G2S2, peak #3—FA2BG2S2, peak #4 FA2[3]G1S1, peak #6—FA2G2S1, peak #13—FA2G2 and peak #14—FA2BG2). The peak distributions in all examined groups are summarized in Table 2 with the corresponding structures. Representative comparisons are depicted in Figure 2.

### 2.4. IgG N-Glycosylation of Infants Born to Normal Weight and Obese Mothers with or Without GDM

**Figure 3:**
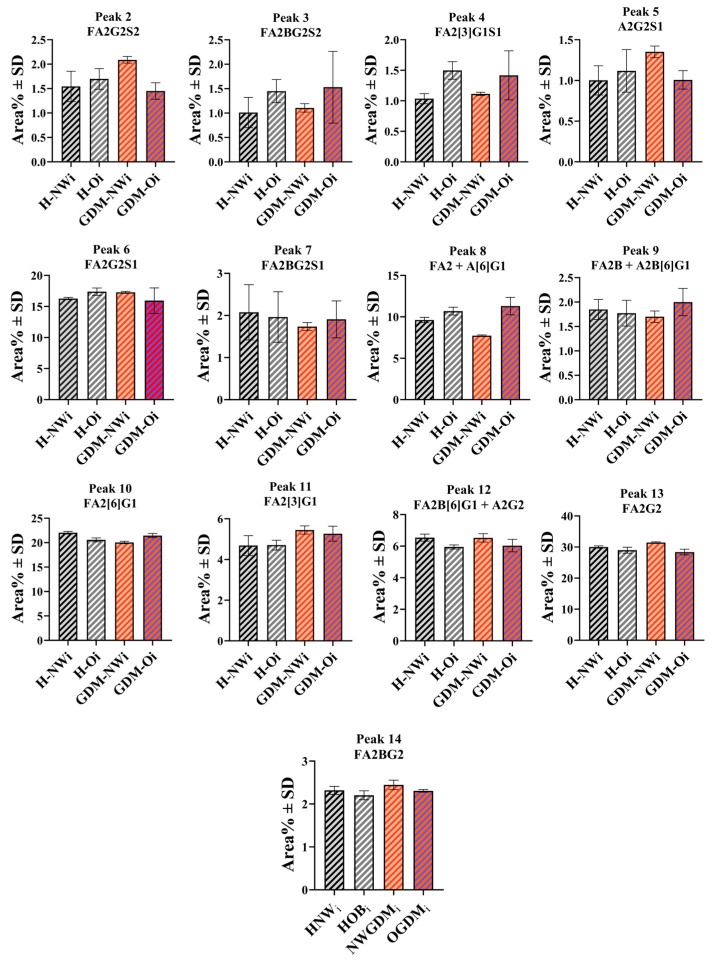
Variation in IgG N-glycan structures across the infant groups. The samples were pooled before the IgG specific N-glycans were prepared and analyzed by CE-LIF. The columns represent mean values, and the error bars indicate standard deviations (SD), both calculated as described above for the pooled infant samples, in the same way as for the maternal pooled samples and as indicated in the legend of Figure 2. H-NWi: infants of H-NW mothers, H-Oi: infants of H-O mothers, GDM-NWi: infants of GDM-NW mothers, GDM-Oi: infants of GDM–O mothers.

Among the 13 N-linked oligosaccharide structures with the average relative peak area greater than 1% in infant serum samples, nine exhibited notable differences in relative peak areas between the groups. Area percentages of three peaks (peak #4—FA2[3]G1S1, peak #10—FA2[6]G1, peak #12—FA2B[6]G1 + A2G2) differed in the H-NW and H-O groups, while the GDM-NW group showed distinct relative abundances compared to the H-NW group in four structures (peak #2—FA2G2S2, peak #5—A2G2S1, peak #10—FA2[6]G1 and peak #11—FA2[3]G1). The relative distribution of the detected and identified glycan structures across the groups are shown in Figure 3.

### 2.5. Sialo-Form to Neutral Form Ratios (SF/NF) in the IgG N-Glycosylation Patterns

Sialylation of IgG modulates its inflammatory response properties by affecting its binding to FcγR receptors, C1q and DC-SIGN [35,36]. The SF/NF ratio in maternal and infant IgG was calculated as described in the Statistical Analysis Section 4.7

The ratio of sialylated structures to neutral ones (SF/NF) was similar in the GDM-NW mothers (0.33 ± 0.02) and the H-NW group (0.33 ± 0.01), while the H-O (0.29 ± 0.02) and GDM-O (0.32 ± 0.02) groups showed slightly decreasing tendency compared to the H-NW group.

In infants, SF/NF ratios were higher in those born to H-O mothers (0.34 ± 0.02) compared to infants of H-NW (0.30 ± 0.03) and GDM-O mothers (0.30 ± 0.01). On the other hand, infants of GDM-O mothers showed a slight decreasing tendency compared to infants of GDM-NW mothers (0.33 ± 0.00).

**Figure 4:**
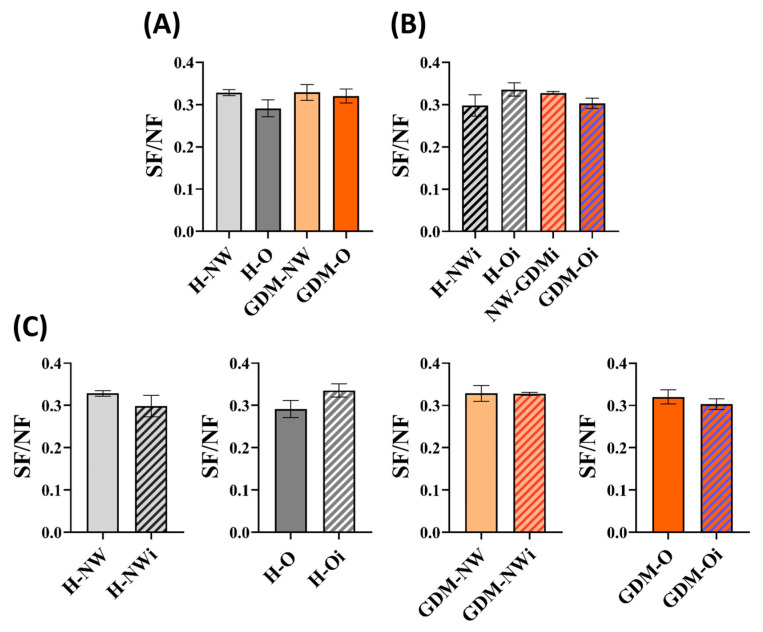
Analysis of SF/NF ratios in the maternal and infant cohorts. (**A**) Maternal groups (**B**) Infant groups, (**C**) Comparative analysis of maternal versus infant SF/NF ratios. Maternal groups: H-NW—healthy normal weight, H-O—healthy obese, GDM-NW—GDM with normal weight, GDM-O—GDM with obesity; Infant groups: H-NWi—infants of healthy normal weight mothers, H-Oi—infants of healthy obese mothers, GDM-NWi—infants of GDM mothers with normal weight, GDM-Oi—infants of GDM mothers with obesity.

Comparisons between the SF/NF ratios of maternal and offspring IgG N-linked glycosylation revealed observable difference in H-NW as well as H-O groups, suggesting that the maternal metabolic status may influence infant IgG sialylation. The SF/NF data corresponding to each maternal and infant group are presented in Table 2 and Figure 4.

### 2.6. Ratio of Core Fucosylation to the Total N-Glycosylation (CF/TNG) Calculated for Maternal and Fetal Serum IgG

It is broadly reported that core fucosylation at Asn297 in the IgG Fc region modulates binding to FcγRIIIa and affects ADCC activity [37].

Maternal IgG CF/TNG value showed a slightly increasing tendency in H-O (0.98 ± 0.00) and GDM-O (0.98 ± 0.00) groups compared to the H-NW (0.97 ± 0.00) and GDM-NW (0.97 ± 0.00).

The CF/TNG values were consistent across all infant groups (0.99 ± 0.01), independent of maternal BMI and GDM status.

**Figure 5:**
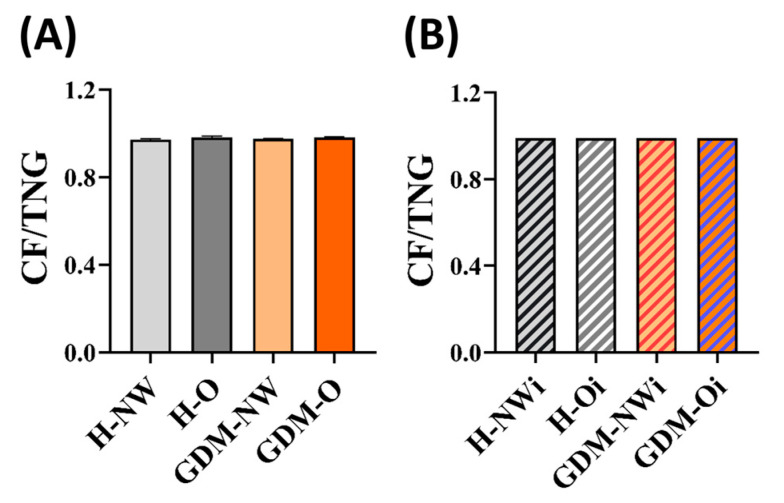
CF/TNG ratios in the maternal and infant groups. (**A**) Maternal groups. (**B**) Infant group. Maternal groups: H-NW—healthy normal weight, H-O—healthy obese, GDM-NW—GDM with normal weight, GDM-O—GDM with obesity; Infant groups: H-NWi—infants of healthy normal weight mothers, H-Oi—infants of healthy obese mothers, GDM-NWi—infants of GDM mothers with normal weight, GDM-Oi—infants of GDM mothers with obesity.

These observations suggest that maternal obesity may be associated with subtle alterations in IgG core fucosylation, while infant IgG CF/TNG ratios appear largely unaffected. Maternal and infant IgG core fucosylation levels relative to total N-glycosylation (CF/TNG) are presented in Table 2 and Figure 5.

## 3. Discussion

This study aimed to provide an exploratory characterization of maternal serum IgG N-glycosylation patterns associated with gestational diabetes mellitus (GDM) and obesity, using CE-LIF analysis. Since IgG crosses the placenta and is detected in infant serum [38], we also examined infant serum IgG N-glycans to explore potential associations of maternal metabolic conditions with placental transport.

We identified 16 and 15 IgG-associated N-glycan structures with mean relative area percentages exceeding 1% across the maternal and infant groups, respectively. Differences were observed in glycosylation traits—including sialylation (SF/NF ratio), core fucosylation (CF/TNG ratio), and individual glycan peaks—between groups of mothers and their infants. Notably, normal and obese mother-infant pairs exhibited differences in the sialylation degree of IgG antibodies, which likely reflects a selective placental transport pattern, whereas such a pattern was not observed in GDM cases.

### 3.1. Maternal IgG N-Glycosylation

Our results indicate that both obesity and GDM are associated with specific maternal IgG N-glycosylation patterns. In particular, GDM-NW mothers could be distinguished from controls by the relative area percentage of four glycans (peak #9—FA2B + A2B[6]G1 and peak # 12—FA2B[6]G1 + A2G2, while healthy obese (H-O) and GDM mothers with obesity (GDM-O) differed in four peaks corresponding to structures of peak #6—FA2G2S1, peak #8—FA2 + A[6]G1, peak #10—FA2[6]G1 and peak #11—FA2[3]G1. These exploratory observations are broadly consistent with our work although the current postpartum sampling design makes direct comparison more complex. In our previous study, conducted at delivery, we were unable to identify structures differentiating the obese from GDM obese mothers [39]. Differences between the studies likely reflect the timing of sample collection: whereas our earlier analysis captured acute effects of GDM and obesity at delivery, the present postpartum (three months) samples may reflect a reversion toward pre-pregnancy glycan profiles. This interpretation is in line with the findings of Bondt et al. [40], who reported trimester-dependent IgG N-glycosylation modifications using MALDI-TOF-MS technique. They found that the extent of sialylation gradually increased during pregnancy and then significantly decreased after delivery. The glycan profiles of IgG isolated from samples collected at 12 and 26 weeks postpartum were substantially different from those detected in the first, second, and third trimesters highlighting the dynamic nature of glycosylation in relation to maternal physiological state [40]. Considering their results, our postpartum samples may therefore represent characteristic IgG N-glycan profile closer to the pre-pregnancy or early gestational state, rather than disease-specific signatures.

Obesity had a stronger association with maternal IgG glycosylation than GDM. While multiple glycans differed between obese and normal-weight mothers, only one glycan (peak #14—FA2BG2) differed between the GDM and non-GDM groups, suggesting that obesity has a more pronounced effect than GDM, and that GDM does not further exacerbate obesity-associated changes.

Consistent with other previously reported data [31], we observed notable differences in the distribution of several N-glycan structures between the H-NW and H-O maternal groups. Among the four structures that exhibited differences (peak #1—A2G2S2, peak #2—FA2G2S2, peak #3—FA2BG2S2 and peak #10—FA2[6]G1), the sialylated glycans were A2G2S2, FA2G2S2, FA2BG2S2 (Figure 2). The reduction in their relative abundance in the obese group may reflect obesity-related modulation of IgG glycosylation. Notably, the relative area percentage value of the FABG2S2 structure (peak #3) showed pronounced variations between control and H-O groups, as well as between the control H-NW and GDM-O groups. These observations should be considered descriptive, requiring confirmation in larger, individual-level studies before any functional relevance can be determined.

### 3.2. Infant IgG N-Glycosylation

The exploratory analysis of infant-derived IgG antibodies revealed that both maternal obesity and gestational diabetes mellitus (GDM) may be associated with differences in infant N-glycosylation profile. We were able to differentiate infants born to mothers with normal BMI (H-NWi) from those born to obese mothers (H-Oi) based on the relative abundance of four N-glycan structures: peak #9—FA2[3]G1S1, peak #10—FA2[6]G1 and peak #12—FA2B[6]G + A2G2. Additionally, differences were also apparent between the H-NWi and GDM-NWi groups in the relative abundance of four structures (peak #2—FA2G2S2, peak #5—A2G2S1, peak #10—FA2[6]G1 and peak #11—FA2[3]G1. However, infants of obese mothers with (GDM-Oi) and without GDM (H-Oi) displayed broadly similar glycosylation profiles. This suggests that maternal obesity may exert a stronger influence on infant IgG N-glycan profile than GDM alone. Unfortunately, to the best of our knowledge, no similar studies have been conducted to date, which limits our ability to compare our observations with other research. The trends observed when comparing H-Oi and GDM-Oi infants appear consistent with our previous maternal findings, as no notable differences were apparent between obese mothers and those with obesity plus GDM [39]. Differences observed between H-Oi and GDM-NWi (peak #8—FA2 + A[6]G1, peak #11—FA2[3]G1, peak #12—FA2B[6]G + A2G2, peak #13—FA2G2, and peak #14—FA2BG2), as well as among the two GDM groups (peak #2—FA2G2S2, peak #5—A2G2S1, peak #8—FA2 + A[6]G1, peak #10—FA2[6]G1 and peak #13—FA2G2), may suggest that maternal metabolic status could influence the post translational modifications of IgG antibodies transferred to infants. The mechanisms and functional significance of this observations remain unclear, and the patterns may reflect, at least in part, selective placental transfer, although Fc and Fab glycans were not separated, and no direct transport measurements were performed. Given the pooled nature of the samples and the limited cohort size, these findings should be interpreted as descriptive and exploratory rather than definitive. Future studies with individual-level data and more detailed clinical characterization will be required to further investigate these preliminary observations and clarify their biological relevance.

### 3.3. Serum IgG N-Glycosylation Properties

#### 3.3.1. Maternal IgG

The reduction in IgG sialylation, which may be linked to an increase in IgG pro-inflammatory activity due to enhanced binding to the FcγR receptor and C1q, was also reported in prior studies [35,36]. In line with our previous report [39], no significant differences in SF/NF ratios were observed between the control H-NW and H-O groups; however, in the present study, the H-O group consistently showed a non-significant trend toward reduced sialylation compared with other groups. Interestingly, this trend was most pronounced relative to the GDM-NW group, which displayed the highest SF/NF ratio. These results emphasize the need for further research involving a larger sample size, especially given that recent findings indicated that hyposialylated IgG, associated with obesity, acted as a ligand for FcγRIIB in endothelial cells, thereby playing a crucial role in obesity-induced insulin resistance [29].

Analysis of the maternal groups revealed a modest increase in core fucosylation in the H-O and GDM-O groups relative to the H-NW group. The findings indicate that the core fucosylation of IgG may be influenced primarily by maternal obesity in this cohort. This aligns with previous reports, which demonstrated that higher levels of core fucosylation in IgG are linked to elevated risk of central adiposity in individuals with normal BMI [32]. In contrast to prior work documenting a cardiovascular-protective role of the FA2[3]G1 structure—including reports of an increased prevalence of this structure among formerly sedentary obese individuals after starting regular exercise [41]—we observed a decreasing trend in the relative area percentage of FA2[3]G1 structure (peak #11) in H-O, GDM-NW, and GDM-O mothers compared with H-NW. This apparent discrepancy may reflect differences in study populations, sampling time points (postpartum versus during/after interventions), or methodological factors (including pooling). Given the descriptive nature of our data, the functional and clinical significance of the observed FA2[3]G1 variation remains uncertain and could warrant further investigation in larger cohorts with individual-level analyses.

#### 3.3.2. Maternal and Infant IgG N-Glycosylation Properties

As the production of IgG antibodies in human infants typically begins later than the studied period [42], the infant IgG N-glycan profiles in our dataset likely reflect the glycosylation pattern of maternal antibodies transferred through the placenta. These pooled samples provide preliminary insights into how maternal metabolic status might be associated with IgG transfer characteristics. In particular, trends in sialylation appeared to differ between the normal-weight and obese groups. While previous reports on maternal-infant IgG glycosylation relationships are inconsistent [43,44,45,46], we observed possible reduction in the sialylation level in H-NWi compared to the H-NW, although its biological significance remains unclear. It is essential to highlight that the data reported in this study represented the N-glycome of intact IgG molecules, without Fc and Fab partitioning. Since only 15–25% of the Fab region carries N-glycan modifications [47], whereas the Fc region is consistently glycosylated, the observed patterns mostly reflect the Fc glycans. Given the lack of Fc/Fab specification and direct transport data, the extent to which glycosylation modulates placental IgG transfer remains uncertain. Within the limitations of our pooled dataset, core fucosylation (CF/TNG ratio) did not show notable differences across mother–infant pairs.

### 3.4. Study Limitations

Due to the young age of the children (3-month-old infants), only a small amount of serum samples could be collected, so the infant samples were pooled before analysis. Maternal samples were also pooled due to logistical and resource constraints, including the limited time and materials available for a comprehensive study in addition to the small sample groups (*n* = 15). Since the samples were pooled statistical analyses of IgG glycosylation patterns could not be performed, so individual variability within the groups could not be assessed.

Another limitation of the study was that, unfortunately, the Ukrainian healthcare system did not allow determination of CRP levels of the subjects included in the study. Therefore, we cannot support the presence of inflammation in these subjects using the parameters currently commonly used in clinical practice.

Additionally, maternal age may act as a potential confounder. Although no statistically significant differences were observed between the groups, the approximately five-year age gap may still be clinically relevant. Given the small sample size and the pooled nature of the glycosylation analysis, age-related effects on IgG N-glycosylation and pregnancy outcomes cannot be excluded. Future studies with larger cohorts and individual-level data should consider maternal age to better evaluate its potential impact.

## 4. Materials and Methods

### 4.1. Samples

Screening for GDM among pregnant women in the high-risk group (e.g., BMI ≥ 25, age ≥ 30) was performed between 12 and 16 weeks of pregnancy using the oral glucose tolerance test (OGTT). High-risk status was defined by the presence of one or more established criteria: previous hyperglycemia in pregnancy; previously elevated blood glucose levels; maternal age ≥ 40 years; high-risk ethnicity; first-degree family history of diabetes; pre-pregnancy BMI > 30 kg/m^2^; previous macrosomia; polycystic ovarian syndrome; or use of corticosteroids/antipsychotics. Pregnant women in the low-risk group for GDM (normal pre-pregnancy BMI, age < 40 years, no previous hyperglycemia in pregnancy, no family history of diabetes, and no other established risk factors) were screened for GDM between 24 and 28 weeks using OGTT. When early screening in high-risk women was negative, routine OGTT at 24–28 weeks was also performed. The reference values recommended by the World Health Organization (WHO) were used for the diagnosis of GDM. Patients who met at least one of the following criteria were classified into the GDM group: (1) fasting plasma glucose = 5.1–6.9 mmol/L (92–125 mg/dL); (2) 1-h post 75 g oral glucose load ≥ 10.0 mmol/L (180 mg/dL), (3) 2-h post 75 g oral glucose load 8.5–11.0 mmol/L (153–199 mg/dL) [48]. The WHO classification of overweight and obesity was used to further categorize the samples (Table 3).

Subjects were excluded from the study if: (1) the mother had type 1 diabetes mellitus; (2) the mother was on a special diet; (3) a genetic disorder had been identified in the mother and/or unborn child; and (4) twin pregnancy. Blood samples were collected from mothers and their infants who met the study criteria at 12 weeks postpartum, in accordance with ethical permit No. 6 dated 16 November 2018, at the Lviv City Children’s Clinical Hospital, Lviv, Ukraine. All participants were of Ukrainian ethnicity (white Caucasian), with no inter-ethnic variation within the study groups. Written informed consent to participate the study was obtained from all participants. The consent form was approved by the institutional ethics board and includes consent for the medical procedures described in the study. No identifiable information is included in the publication. The collected samples were classified into four groups according to maternal BMI and the presence or absence of gestational diabetes mellitus (GDM), and the corresponding infant groups were defined accordingly:

(1) **Healthy normal weight (H-NW)**: women with normal BMI (18.5–24.9 kg/m^2^) and uncomplicated pregnancy, without GDM or other complications; infants of H-NW mothers (**H-NWi**)
(2) **Healthy obese (H-O)**: women with obesity (BMI ≥ 30 kg/m^2^) and uncomplicated pregnancy, without GDM; infants of H-O mothers (**H-Oi**):
(3) **GDM with normal weight (GDM-NW)**: women with normal BMI and gestational diabetes mellitus; infants of GDM-NW (**GDM-NWi**)
(4) **GDM with obesity (GDM-O)**: women with obesity and gestational diabetes mellitus; infants of GDM-O (**GDM-Oi**)

Each group contained 15 mother-child pairs. Whole blood samples were collected into glass tubes with graduations (PrAT “Skloprilad”, Poltava, Ukraine), and the clots were removed by centrifuging the tubes at 1500× *g* for 10 min at room temperature (22–23 °C). The separated serum samples were transferred into 1.5 mL microvials and stored at −80 °C until analysis.

Prior to analysis, serum samples from each maternal and infant group were pooled to obtain sufficient volume for CE-LIF measurement. Each pool was then analyzed in technical triplicates. This pooling strategy was applied consistently across all groups.

### 4.2. Chemicals

The following chemicals and reagents were used in all experimental procedures: sodium dodecyl sulfate (SDS) and acetonitrile (ACN) were from VWR (Radnor, PA, USA). The endoglycosidase PNGase F was from Asparia Glycomics (San Sebastián, Spain). The Fast Glycan Labeling and Analysis Kit, including the 8-aminopyrene-1,3,6-trisulfonic acid (APTS) labeling dye, the magnetic beads for purification, the maltooligosaccharide ladder, and the maltose internal standard, as well as the N-linked carbohydrate separation gel buffer (NCHO), was purchased from Bio-Science Kft. (Budapest, Hungary). Sodium cyanoborohydride (1 M in tetrahydrofuran), glycerol, dithiothreitol (DTT), sodium bicarbonate (NaHCO_3_) were obtained from Sigma Aldrich (St. Louis, MO, USA). Protein G microaffinity columns and buffers used for IgG purification were ordered from PhyNexus Inc. (San Jose, CA, USA).

### 4.3. IgG Partitioning

**Figure 6:**
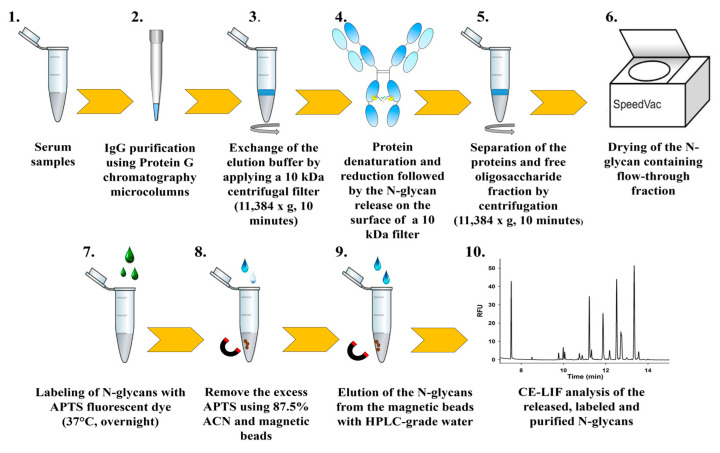
The main purification steps of serum immunoglobulin G. Serum IgG was partitioned using Protein G tips followed by transfer to the top of 10 kDa spinfilters, where the elution buffer was removed by centrifugation. Protein denaturation and reduction, as well as the N-glycan-release steps were performed on the filters. The liberated N-glycans were separated from the polypeptide backbone by centrifugation, then dried and labeled with APTS fluorescence dye. Excess dye was removed by using magnetic beads and washing the samples with ACN. Finally, the purified APTS-labeled N-glycan contents of the IgGs were analyzed with a CE-LIF as described below. This workflow enabled the detailed profiling of IgG N-glycosylation in maternal and infant serum samples, allowing us to explore potential alterations associated with maternal metabolic status and gestational diabetes.

The Protein G PhyTip^®^ columns (1 mL volume) containing 40 μL affinity media per column were conditioned with 200 μL of Capture/Wash 1 Buffer containing 10 mM NaH_2_PO_4_, 140 mM NaCl (pH 7.4) before applying 400 μL per column of a 1:1 mixture of sample and Capture/Wash 1 Buffer. After the IgG binding step, the resin was washed twice with 200 μL of Capture/Wash 1 Buffer, then once with 200 μL of Wash 2 Buffer (140 mM NaCl) followed by elution of the bound antibodies using 200 μL of freshly prepared 10% acetic acid. The partitioning steps of IgG from serum samples are briefly illustrated in Figure 6.

### 4.4. N-Glycan Release, Fluorophore Labeling and Preparation for CE-LIF Analysis

After the partitioning step, the elution buffer was removed by using 10 kDa centrifugal filters and centrifuging the samples at 11,384× *g* for 10 min. Then, the filters were washed twice with 50 μL of HPLC-grade water (11,384× *g*; 10 min). Protein denaturation and reduction were performed (80 °C; 10 min) on the filter by adding a mixture of 10 μL of HPLC-grade water and 4 μL of denaturation solution (400 mM DTT and 5% SDS). After removing the denaturing solution by centrifugation, the filter was rewashed with 30 μL of water. The N-glycan contents of the proteins were released by the addition of a mixture of 49 μL of 20 mM NaHCO_3_ (pH 7.0) and 1 μL of PNGase F (200 mU) incubated at 37 °C overnight. The released glycans were washed away from the filter with 30 μL of HPLC-grade water and dried in a vacuum centrifuge (Thermo Scientific, Schaumburg, IL, USA). The sugars to be analyzed were labeled with a reaction mixture of 6 μL of 20 mM 8-aminopyrene-1,3,6-trisulfonic acid (APTS) (dissolved in 15% acetic acid) and 2 μL of 1 M sodium cyanoborohydride (NaBH_3_CN) solution (1 M in tetrahydrofuran) by incubation at 37 °C overnight. The excess dye was removed from the labeled samples by magnetic beads [50], and the samples were washed three times with 87.5% ACN.

### 4.5. Analysis of the Labeled N-Glycans Using Capillary Electrophoresis with Laser-Induced Fluorescence Detection (CE-LIF)

Capillary electrophoresis analyses were performed using a PA800 ProteomeLab Protein Characterization System (Beckman Coulter Inc., Brea, CA, USA) equipped with a laser-induced fluorescence detector. To detect the N-glycan content of the samples, the APTS tagging dye was excited with a 488 nm laser beam, and the emitted light was collected using a 520 nm emission filter, built into the stock Photomultiplier Tube (PMT) unit of the CE instrument. A bare fused silica (BFS) (Optronis GmbH, Kehl, Germany) capillary with an effective length of 50 cm (60 cm full length) and an internal diameter of 50 μm was used for the separations. The capillary was filled with N-linked carbohydrate separation gel buffer (NCHO, pH 4.75) before sample injection. During the separation process, the cathode was placed at the injection side, while the anode at the detection side. The temperature of the capillary cassette was set at 25 °C and a voltage of 30 kV was applied during the analyses. The samples were pressure-injected at 1 psi (6.89 kPa) for 5 s. APTS-labeled maltose was used as an internal standard and was injected at 1 psi for 5 s before the samples.

### 4.6. Data Analysis

32Karat (version 7.0) software package from Beckman Coulter was utilized for data acquisition and processing. The GU values of the peaks, crucial for identifying N-glycan structures, were determined by using the GUcal Software (version 1.1b) (University of Pannonia, Veszprem, Hungary, https://hlbs.org/index.php/gucal (accessed on 15 September 2024)). N-glycan structures were assigned to the peaks based on literature data and by using the built-in database of the GUcal software. Peaks in the electropherograms with an average relative area reaching a minimum value of 1% (i.e., an area percentage ≥ 1%) were considered essential for identifying the N-glycan content of the examined samples. A relative peak area threshold of 1% was applied to focus the analysis on N-glycan structures that are consistently detectable and quantitatively meaningful across the samples. Peaks below this level may represent minor components or background signals, which are more susceptible to analytical noise and less likely to contribute to biologically relevant differences. The 1% cutoff ensured that only robust and reproducible signals were considered for downstream analysis and biological interpretation, thereby improving data reliability while minimizing the influence of low-abundance, potentially spurious peaks. The normalized peak area percent values of the separated components were calculated using PeakFit v4.12 software (SeaSolve Software Inc., San Jose, CA, USA). All experiments were done in triplicates, and the obtained data are presented as mean ± standard deviation (SD) in the text, tables, and figures.

### 4.7. Statistical Analysis

For statistical analysis, the GraphPad Prism version 8.0.1 (GraphPad Software, San Diego, CA, USA) was used. Data distribution was examined using the Shapiro–Wilk normality test. If the data showed a normal distribution, one-way ANOVA test was employed for multiple comparisons. In case of non-normal distribution, the Kruskal–Wallis test was applied.

The average maternal age and the gestational age were analyzed with one-way ANOVA test, and no differences were found between the groups. Kruskal–Wallis and Dunn’s test were used for the comparison of the maternal BMI. Differences between the groups were considered significant at *p* ≤ 0.05, and denoted by asterisks as follows: * *p* ≤ 0.05; ** *p* ≤ 0.01; *** *p* ≤ 0.001; **** *p* ≤ 0.0001.

The N-glycosylation analysis was performed on technical replicates of pooled samples. Each maternal and infant group represented a single pooled sample (*n* = 1), and the replicates reflected repeated sample processing (three times) and measurement (three times), rather than independent biological samples. Infant samples were pooled due to the limited volume of serum available from 3-month-old subjects. Maternal samples were pooled to accommodate constraints related to sample availability and project resources. Therefore, the N-glycan data are presented descriptively in this paper, without formal statistical comparison between groups.

The Sialo-form to Neutral form ratios (SF/NF) were calculated by dividing the total abundance of sialylated glycans by the total abundance of neutral glycans for each sample. The ratio of core fucosylated glycans to total N-glycans (CF/TNG) was calculated for each maternal and infant sample by dividing the total abundance of core-fucosylated glycans by the total abundance of all N-glycans. This calculation was performed following the previously established method in [51].

Please note, that in this study, we did not include a separate NW (normal weight) group. Instead, we defined an H-NW (healthy normal weight) group, which refers to women with a normal BMI (18.5–24.9 kg/m^2^) and uncomplicated pregnancy (i.e., without gestational diabetes mellitus or other pregnancy complications). The H-NW group served as healthy reference group for all comparisons

## 5. Conclusions

Our findings suggest that maternal obesity and GDM are associated with postpartum differences in IgG N-glycosylation patterns in both mothers and their infants. Observed glycosylation alterations may represent patterns of interest for further investigation, highlighting the need for studies with larger cohorts and detailed clinical characterization. These exploratory observations may provide a basis for follow-up research into maternal and infant immune profiles and their potential functional or clinical implications. Future studies could investigate the relationships between maternal and infant IgG N-glycosylation patterns and maternal metabolic status, as well as explore the potential functional consequences of the observed alterations. Additionally, the development of optimized sampling protocols and longitudinal analyses could help better capture these dynamics over time.

## Figures and Tables

**Table 1:** Demographic and clinical data of the participants involved in the experiments.

Group	Age of Mothers (Mean Year ± SD)	Maternal BMI (kg/m^2^) (Mean ± SD)	Gestational Age (Weeks ± SD)	Delivery Mode in % (Natural/Cesarean-Section)
Healthy Normal Weight (H-NW, *n* = 15).	26.47 ± 4.98 ^ns^	22.75 ± 1.21	38.94 ± 1.91	80%/20%
Healthy Obese (H-O, *n* = 15)	26.20 ± 5.27 ^ns^	31.36 ± 1.43 ^a,^****	37.96 ± 1.41 ^b,^**	86.66%/13.34%
GDM + Normal Weight (GDM-NW, *n* = 15)	27.87 ± 5.19 ^ns^	23.07 ± 1.83	38.59 ± 0.88	53.34%/46.66%
GDM + Obese (GDM-O, *n* = 15)	30.73 ± 7.21 ^ns^	32.49 ± 2.15 ^a,^****	39.96 ± 1.66 ^a,^**	73.34%/26.66%

Maternal and child serum IgG antibodies were purified using affinity chromatography columns as detailed in the procedure outlined below. Mothers with a BMI ≥ 30 were classified into the obese groups and patients with a BMI ≤ 30 into the normal weight groups according to the WHO criteria [33]. One-way ANOVA was used to examine the average age of the maternal groups and Mann–Whitney test was applied for the analysis of maternal BMI. The Kruskal–Wallis test was used for the statistical analysis of any other patient characteristics. ns: non-significant, ** *p* ≤ 0.01, **** *p* ≤ 0.0001. ^a^: significant difference between the control (H-NW) and the indicated groups; ^b^: significant difference between the H-O and GDM-O groups.

**Table 2:** Percentage of the relative peak area of N-glycan structures identified in IgG samples partitioned from maternal and infant serum and the calculated degree of sialylation (SF/NF ratio: ratio of sialylated structures to non-sialylated ones) and the ratio of core fucosylation to the total N-glycosylation of IgGs (CF/TNG).

Peak No.	N-Glycan	GU-Value	Sample	Groups				Peak No.	N-Glycan	GU-Value	Sample	Groups			
				H-NW	H-O	GDM-NW	GDM-O					H-NW	H-O	GDM-NW	GDM-O
				Mean Area % ± SD								Mean Area % ± SD			
**1**	A2G2S2	4.62 ± 0.01	Maternal	1.46 ± 0.05	0.52 ± 0.07	1.27 ± 0.16	0.79 ± 0.22	**8**	FA2; A[6]G1	7.77 ± 0.03	Maternal	12.02 ± 0.29	12.45 ± 0.25	10.44 ± 0.27	10.32 ± 0.30
			Infants	N/A	N/A	N/A	N/A				Infants	9.62 ± 0.32	10.69 ± 0.47	7.73 ± 0.09	11.30 ± 1.05
**2**	FA2G2S2	4.90 ± 0.01	Maternal	2.43 ± 0.06	1.59 ± 0.17	2.27 ± 0.10	1.91 ± 0.28	**9**	FA2B; A2B[6]G1	8.29 ± 0.03	Maternal	2.56 ± 0.07	2.22 ± 0.26	1.78 ± 0.18	2.16 ± 0.07
			Infants	1.55 ± 0.31	1.70 ± 0.21	2.09 ± 0.07	1.45 ± 0.17				Infants	1.85 ± 0.20	1.77 ± 0.26	1.70 ± 0.12	2.00 ± 0.28
**3**	FA2BG2S2	5.00 ± 0.01	Maternal	1.61 ± 0.04	1.18 ± 0.24	1.41 ± 0.14	1.19 ± 0.17	**10**	FA2[6]G1	8.85 ± 0.03	Maternal	20.27 ± 0.39	21.41 ± 0.57	20.96 ± 0.25	20.41 ± 0.32
			Infants	1.01 ± 0.31	1.45 ± 0.24	1.11 ± 0.09	1.53 ± 0.73				Infants	22.03 ± 0.28	20.57 ± 0.39	20.02 ± 0.25	21.44 ± 0.41
**4**	FA2[3]G1S1	5.98 ± 0.02	Maternal	1.45 ± 0.12	1.48 ± 0.55	1.27 ± 0.07	1.22 ± 0.01	**11**	FA2[3]G1	9.18 ± 0.03	Maternal	6.86 ± 0.35	6.23 ± 0.19	6.30 ± 0.19	6.36 ± 0.55
			Infants	1.04 ± 0.08	1.50 ± 0.14	1.11 ± 0.03	1.42 ± 0.40				Infants	4.69 ± 0.49	4.71 ± 0.24	5.45 ± 0.20	5.27 ± 0.37
**5**	A2G2S1	6.19 ± 0.02	Maternal	1.12 ± 0.07	1.20 ± 0.49	1.31 ± 0.21	1.03 ± 0.15	**12**	FA2B[6]G; A2G2	9.23 ± 0.03	Maternal	6.26 ± 0.26	5.89 ± 0.16	5.57 ± 0.29	5.72 ± 0.44
			Infants	1.00 ± 0.18	1.12 ± 0.26	1.35 ± 0.07	1.01 ± 0.11				Infants	6.54 ± 0.22	5.95 ± 0.13	6.52 ± 0.27	6.03 ± 0.40
**6**	FA2G2S1	6.71 ± 0.02	Maternal	14.20 ± 0.16	14.10 ± 0.45	15.14 ± 0.31	15.51 ± 0.21	**13**	FA2G2	10.22 ± 0.04	Maternal	25.30 ± 0.32	26.99 ± 0.92	27.75 ± 0.34	28.32 ± 0.09
			Infants	16.28 ± 0.19	17.39 ± 0.58	17.28 ± 0.17	15.94 ± 2.04				Infants	30.02 ± 0.37	28.98 ± 0.96	31.45 ± 0.23	28.40 ± 0.91
**7**	FA2BG2S1	6.87 ± 0.02	Maternal	2.30 ± 0.09	2.46 ± 0.36	2.39 ± 0.27	2.59 ± 0.07	**14**	FA2BG2	10.57 ± 0.04	Maternal	2.15 ± 0.03	2.28 ± 0.09	2.15 ± 0.14	2.48 ± 0.09
			Infants	2.08 ± 0.66	1.96 ± 0.60	1.74 ± 0.09	1.91 ± 0.44				Infants	2.32 ± 0.09	2.20 ± 0.11	2.45 ± 0.11	2.31 ± 0.03
**SF/NF ratio**			Maternal	0.33 ± 0.01	0.29 ± 0.02	0.33 ± 0.02	0.32 ± 0.02	**CF/TNG ratio**			Maternal	0.97 ± 0.00	0.98 ± 0.00	0.97 ± 0.00	0.98 ± 0.00
			Infants	0.30 ± 0.03	0.34 ± 0.02	0.33 ± 0.00	0.30 ± 0.01				Infants	0.99 ± 0.00	0.99 ± 0.00	0.99 ± 0.00	0.99 ± 0.00

The peaks are numbered according to the markings depicted on the electropherograms in Figure 1. The abbreviated glycan structure names follow the nomenclature proposed by Harvey et al., including the linkage isomer annotation in brackets [34]. Maternal groups: H-NW—healthy normal weight, H-O—healthy obese, GDM-NW—GDM with normal weight, GDM-O—GDM with obesity; Groups of infants: H-NWi—infants of healthy normal weight, H-Oi—infants of healthy obese, GDM-NWi—infants of GDM women with normal weight, GDM-Oi—infants of GDM women with obesity. N/A: the structure was present but did not reach the threshold; percentage values are not provided.

**Table 3:** WHO classification of obesity based on BMI [49].

Classification	BMI (kg/m2)	Obesity Class
Underweight	<18.5	
Normal	18.5–24.9	
Overweight	25.0–29.9	
Obesity	30.0–34.9	I
	35.0–39.9	II
Extreme Obesity	≥40	III

## Data Availability

The data presented in this study are available on request from the corresponding author. Data are unavailable to be published due to privacy and ethical restrictions.

## Abbreviations

The following abbreviations are used in this manuscript:

ACN	acetonitrile
ADCC	antibody-dependent cellular cytotoxicity
APTS	8-aminopyrene-1,3,6-trisulfonic acid
BMI	body mass index
CE-LIF	Capillary electrophoresis with laser-induced fluorescent detection
CF/TNG ratio	ratio of core-fucosylated structures to the total N-glycosylation
DC-SIGN	dendritic cell-specific ICAM-3 grabbing non-integrin
DTT	dithiothreitol
Fc	fragment crystallizable
GDM	gestational diabetes mellitus
GDM-O	GDM with obesity
H-NW	Healthy Normal Weight
H-NWi	infant of healthy normal weight mothers
H-O	Healthy Obese
H-Oi	infant of healthy obese mothers
IgG	immunoglobulin G
NCHO	N-linked carbohydrate separation gel buffer
GDM-NW	GDM with normal weight
GDM-NWi	Infant of GDM with normal weight mothers
GDM-Oi	Infant of GDM with obesity mothers
OGTT	Oral glucose tolerance test
PNGase F	Peptide N-Glycosidase F
RFU	relative fluorescence unit
SDS	sodium dodecyl sulfate
SF/NF ratio	ratio of the sialo form to neutral structures
WHO	World Health Organization

## References

[1] Alberti K.G.M.M., Zimmet P.Z. (1998). Definition, diagnosis and classification of diabetes mellitus and its complications. Part 1: Diagnosis and classification of diabetes mellitus. Provisional report of a WHO Consultation. Diabet. Med..

[2] Abell S.K., De Courten B., Boyle J.A., Teede H.J. (2015). Inflammatory and Other Biomarkers: Role in Pathophysiology and Prediction of Gestational Diabetes Mellitus. Int. J. Mol. Sci..

[3] World Health Organization (2014). Diagnostic criteria and classification of hyperglycaemia first detected in pregnancy: A World Health Organization Guideline. Diabetes Res. Clin. Pract..

[4] American Diabetes Association (2020). 14. Management of Diabetes in Pregnancy: Standards of Medical Care in Diabetes-2020. Diabetes Care.

[5] National Institute for Health and Care Excellence (2020). Diabetes in Pregnancy: Management from Preconception to the Postnatal Period.

[6] Schafer-Graf U.M., Gembruch U., Kainer F., Groten T., Hummel S., Hosli I., Grieshop M., Kaltheuner M., Buhrer C., Kautzky-Willer A. (2018). Gestational Diabetes Mellitus (GDM)—Diagnosis, Treatment and Follow-Up. Guideline of the DDG and DGGG (S3 Level, AWMF Registry Number 057/008, February 2018). Geburtshilfe Frauenheilkd..

[7] American Diabetes Association Professional Practice Committee (2022). 2. Classification and Diagnosis of Diabetes: Standards of Medical Care in Diabetes-2022. Diabetes Care.

[8] de Wit L., Bos D.M., van Rossum A.P., van Rijn B.B., Boers K.E. (2019). Repeated oral glucose tolerance tests in women at risk for gestational diabetes mellitus. Eur. J. Obstet. Gynecol. Reprod. Biol..

[9] Maor-Sagie E., Hallak M., Toledano Y., Gabbay-Benziv R. (2023). Oral Glucose Tolerance Test Performed after 28 Gestational Weeks and Risk for Future Diabetes-A 5-Year Cohort Study. J. Clin. Med..

[10] Miailhe G., Kayem G., Girard G., Legardeur H., Mandelbrot L. (2015). Selective rather than universal screening for gestational diabetes mellitus?. Eur. J. Obstet. Gynecol. Reprod. Biol..

[11] World Health Organization Obesity and Overweight. https://www.who.int/news-room/fact-sheets/detail/obesity-and-overweight.

[12] Godfrey K.M., Reynolds R.M., Prescott S.L., Nyirenda M., Jaddoe V.W., Eriksson J.G., Broekman B.F. (2017). Influence of maternal obesity on the long-term health of offspring. Lancet Diabetes Endocrinol..

[13] Gaillard R., Felix J.F., Duijts L., Jaddoe V.W. (2014). Childhood consequences of maternal obesity and excessive weight gain during pregnancy. Acta Obstet. Gynecol. Scand..

[14] Neri C., Edlow A.G. (2015). Effects of Maternal Obesity on Fetal Programming: Molecular Approaches. Cold Spring Harb. Perspect. Med..

[15] Aebersold R., Agar J.N., Amster I.J., Baker M.S., Bertozzi C.R., Boja E.S., Costello C.E., Cravatt B.F., Fenselau C., Garcia B.A. (2018). How many human proteoforms are there?. Nat. Chem. Biol..

[16] Pinho S.S., Alves I., Gaifem J., Rabinovich G.A. (2023). Immune regulatory networks coordinated by glycans and glycan-binding proteins in autoimmunity and infection. Cell. Mol. Immunol..

[17] Colley K.J., Varki A., Haltiwanger R.S., Kinoshita T., Varki A., Cummings R.D., Esko J.D., Stanley P., Hart G.W., Aebi M. (2022). Cellular Organization of Glycosylation. Essentials of Glycobiology.

[18] He M., Zhou X., Wang X. (2024). Glycosylation: Mechanisms, biological functions and clinical implications. Signal Transduct. Target Ther..

[19] Taniguchi N., Kizuka Y. (2015). Glycans and cancer: Role of N-glycans in cancer biomarker, progression and metastasis, and therapeutics. Adv. Cancer Res..

[20] Packer N.H., von der Lieth C.W., Aoki-Kinoshita K.F., Lebrilla C.B., Paulson J.C., Raman R., Rudd P., Sasisekharan R., Taniguchi N., York W.S. (2008). Frontiers in glycomics: Bioinformatics and biomarkers in disease. An NIH white paper prepared from discussions by the focus groups at a workshop on the NIH campus, Bethesda MD (11–13 September 2006). Proteomics.

[21] Kam R.K.T., Poon T.C.W. (2008). The Potentials of Glycomics in Biomarker Discovery. Clin. Proteom..

[22] Anthony R.M., Kobayashi T., Wermeling F., Ravetch J.V. (2011). Intravenous gammaglobulin suppresses inflammation through a novel T(H)2 pathway. Nature.

[23] Shields R.L., Lai J., Keck R., O’Connell L.Y., Hong K., Meng Y.G., Weikert S.H., Presta L.G. (2002). Lack of fucose on human IgG1 N-linked oligosaccharide improves binding to human Fcgamma RIII and antibody-dependent cellular toxicity. J. Biol. Chem..

[24] Biermann M.H., Griffante G., Podolska M.J., Boeltz S., Sturmer J., Munoz L.E., Bilyy R., Herrmann M. (2016). Sweet but dangerous—The role of immunoglobulin G glycosylation in autoimmunity and inflammation. Lupus.

[25] Scallon B.J., Tam S.H., McCarthy S.G., Cai A.N., Raju T.S. (2007). Higher levels of sialylated Fc glycans in immunoglobulin G molecules can adversely impact functionality. Mol. Immunol..

[26] Quast I., Keller C.W., Maurer M.A., Giddens J.P., Tackenberg B., Wang L.X., Munz C., Nimmerjahn F., Dalakas M.C., Lunemann J.D. (2015). Sialylation of IgG Fc domain impairs complement-dependent cytotoxicity. J. Clin. Investig..

[27] Abes R., Teillaud J.L. (2010). Impact of Glycosylation on Effector Functions of Therapeutic IgG. Pharmaceuticals.

[28] Lemmers R.F.H., Vilaj M., Urda D., Agakov F., Simurina M., Klaric L., Rudan I., Campbell H., Hayward C., Wilson J.F. (2017). IgG glycan patterns are associated with type 2 diabetes in independent European populations. Biochim. Biophys. Acta Gen. Subj..

[29] Tanigaki K., Sacharidou A., Peng J., Chambliss K.L., Yuhanna I.S., Ghosh D., Ahmed M., Szalai A.J., Vongpatanasin W., Mattrey R.F. (2018). Hyposialylated IgG activates endothelial IgG receptor FcgammaRIIB to promote obesity-induced insulin resistance. J. Clin. Investig..

[30] Stambuk T., Kifer D., Smircic-Duvnjak L., Vucic Lovrencic M., Gornik O. (2023). Associations between plasma protein, IgG and IgA N-glycosylation and metabolic health markers in pregnancy and gestational diabetes. PLoS ONE.

[31] Nikolac Perkovic M., Pucic Bakovic M., Kristic J., Novokmet M., Huffman J.E., Vitart V., Hayward C., Rudan I., Wilson J.F., Campbell H. (2014). The association between galactosylation of immunoglobulin G and body mass index. Prog. Neuropsychopharmacol. Biol. Psychiatry.

[32] Liu D., Li Q., Dong J., Li D., Xu X., Xing W., Zhang X., Cao W., Hou H., Wang H. (2019). The Association Between Normal BMI with Central Adiposity And Proinflammatory Potential Immunoglobulin G N-Glycosylation. Diabetes Metab. Syndr. Obes..

[33] Weir C.B., Jan A. (2024). BMI Classification Percentile And Cut Off Points. StatPearls.

[34] Harvey D.J., Merry A.H., Royle L., Campbell M.P., Rudd P.M. (2011). Symbol nomenclature for representing glycan structures: Extension to cover different carbohydrate types. Proteomics.

[35] Kaneko Y., Nimmerjahn F., Ravetch J.V. (2006). Anti-inflammatory activity of immunoglobulin G resulting from Fc sialylation. Science.

[36] Nimmerjahn F., Anthony R.M., Ravetch J.V. (2007). Agalactosylated IgG antibodies depend on cellular Fc receptors for in vivo activity. Proc. Natl. Acad. Sci. USA.

[37] Golay J., Andrea A.E., Cattaneo I. (2022). Role of Fc Core Fucosylation in the Effector Function of IgG1 Antibodies. Front. Immunol..

[38] Simister N.E., Mostov K.E. (1989). An Fc receptor structurally related to MHC class I antigens. Nature.

[39] Farkas A., Suranyi A., Kolcsar B., Gyurkovits Z., Kozinszky Z., Vari S.G., Guttman A. (2025). Potential Glycobiomarkers in Maternal Obesity and Gestational Diabetes During Human Pregnancy. J. Clin. Med..

[40] Bondt A., Rombouts Y., Selman M.H., Hensbergen P.J., Reiding K.R., Hazes J.M., Dolhain R.J., Wuhrer M. (2014). Immunoglobulin G (IgG) Fab glycosylation analysis using a new mass spectrometric high-throughput profiling method reveals pregnancy-associated changes. Mol. Cell. Proteom..

[41] Simunic-Briski N., Dukaric V., Ocic M., Madzar T., Vinicki M., Frkatovic-Hodzic A., Knjaz D., Lauc G. (2024). Regular moderate physical exercise decreases Glycan Age index of biological age and reduces inflammatory potential of Immunoglobulin G. Glycoconj. J..

[42] Darmawan D., Raychaudhuri S., Lakshminrusimha S., Dimitriades V.R. (2024). Hypogammaglobulinemia in neonates: Illustrative cases and review of the literature. J. Perinatol..

[43] Williams P.J., Arkwright P.D., Rudd P., Scragg I.G., Edge C.J., Wormald M.R., Rademacher T.W. (1995). Short communication: Selective placental transport of maternal IgG to the fetus. Placenta.

[44] Kibe T., Fujimoto S., Ishida C., Togari H., Wada Y., Okada S., Nakagawa H., Tsukamoto Y., Takahashi N. (1996). Glycosylation and Placental Transport of Immunoglobulin G. J. Clin. Biochem. Nutr..

[45] Einarsdottir H.K., Selman M.H., Kapur R., Scherjon S., Koeleman C.A., Deelder A.M., van der Schoot C.E., Vidarsson G., Wuhrer M. (2013). Comparison of the Fc glycosylation of fetal and maternal immunoglobulin G. Glycoconj. J..

[46] Jansen B.C., Bondt A., Reiding K.R., Scherjon S.A., Vidarsson G., Wuhrer M. (2016). MALDI-TOF-MS reveals differential N-linked plasma- and IgG-glycosylation profiles between mothers and their newborns. Sci. Rep..

[47] Stadlmann J., Pabst M., Altmann F. (2010). Analytical and Functional Aspects of Antibody Sialylation. J. Clin. Immunol..

[48] World Health Organization (2013). Diagnostic Criteria and Classification of Hyperglycaemia First Detected in Pregnancy.

[49] National Institutes of Health (1998). Clinical Guidelines on the Identification, Evaluation, and Treatment of Overweight and Obesity in Adults—The Evidence Report. Obes. Res..

[50] Váradi C., Lew C., Guttman A. (2014). Rapid magnetic bead based sample preparation for automated and high throughput N-glycan analysis of therapeutic antibodies. Anal. Chem..

[51] Varadi C., Mittermayr S., Szekrenyes A., Kadas J., Takacs L., Kurucz I., Guttman A. (2013). Analysis of haptoglobin N-glycome alterations in inflammatory and malignant lung diseases by capillary electrophoresis. Electrophoresis.

